# Advances in tumor immune microenvironment of head and neck squamous cell carcinoma: A review of literature

**DOI:** 10.1097/MD.0000000000037387

**Published:** 2024-03-01

**Authors:** Qichao Hong, Shun Ding, Chengliang Xing, Zhonglin Mu

**Affiliations:** a Department of Otorhinolaryngology Head and Neck Surgery, Hainan General Hospital (Hainan Affiliated Hospital of Hainan Medical University), Haikou, China; b Department of Otolaryngology Head and Neck Surgery, The First Affiliated Hospital, Hainan Medical University, Haikou, China.

**Keywords:** head and neck cancers, immune cells, immune checkpoint, immunotherapy, literature review, tumor microenvironment

## Abstract

Squamous cell carcinoma is seen as principal malignancy of head and neck. Tumor immune microenvironment plays a vital role in the occurrence, development and treatment of head and neck squamous cell carcinoma (HNSCC). The effect of immunotherapy, in particular, is closely related to tumor immune microenvironment. This review searched for high-quality literature included within PubMed, Web of Science, and Scopus using the keywords “head and neck cancers,” “tumor microenvironment” and “immunotherapy,” with the view to summarizing the characteristics of HNSCC immune microenvironment and how various subsets of immune cells promote tumorigenesis. At the same time, based on the favorable prospects of immunotherapy having been shown currently, the study is committed to pinpointing the latest progress of HNSCC immunotherapy, which is of great significance in not only further guiding the diagnosis and treatment of HNSCC, but also conducting its prognostic judgement.

## 1. Introduction

Head and neck cancer is regarded as the seventh most common cancer in the world in the light of the Global Cancer Report 2020. With annual new incidence and death rates increasingly growing year by year, head and neck squamous cell cancer carcinoma (HNSCC) is viewed as the most common type yet of cancer.^[1,2]^ The current treatment plan for HNSCC is mainly the comprehensive treatment combined with surgery, radiotherapy and chemotherapy. In recent years, there exist many different ways including traditional Chinese medicine therapy, molecular targeted therapy, immunotherapy and other treatment methods which have gradually been paid tremendous attention by people.^[3]^ In advanced tumors, however, these treatments are often less effective. And the main causes of death in patients suffering from advanced HNSCS are concerned with local recurrence, lymphatic metastases in the neck and standard chemotherapy as well as drug resistance, which all will result in treatment failure.^[4]^ Although patients with the later-stage HNSCC have been treated, the 5-year survival rate for them still remains quite low, at about 40% to 50%.^[5]^ Therefore, it is a key clue to seek an effective therapeutic target or prognostic biomarker.

**Figure 1.**
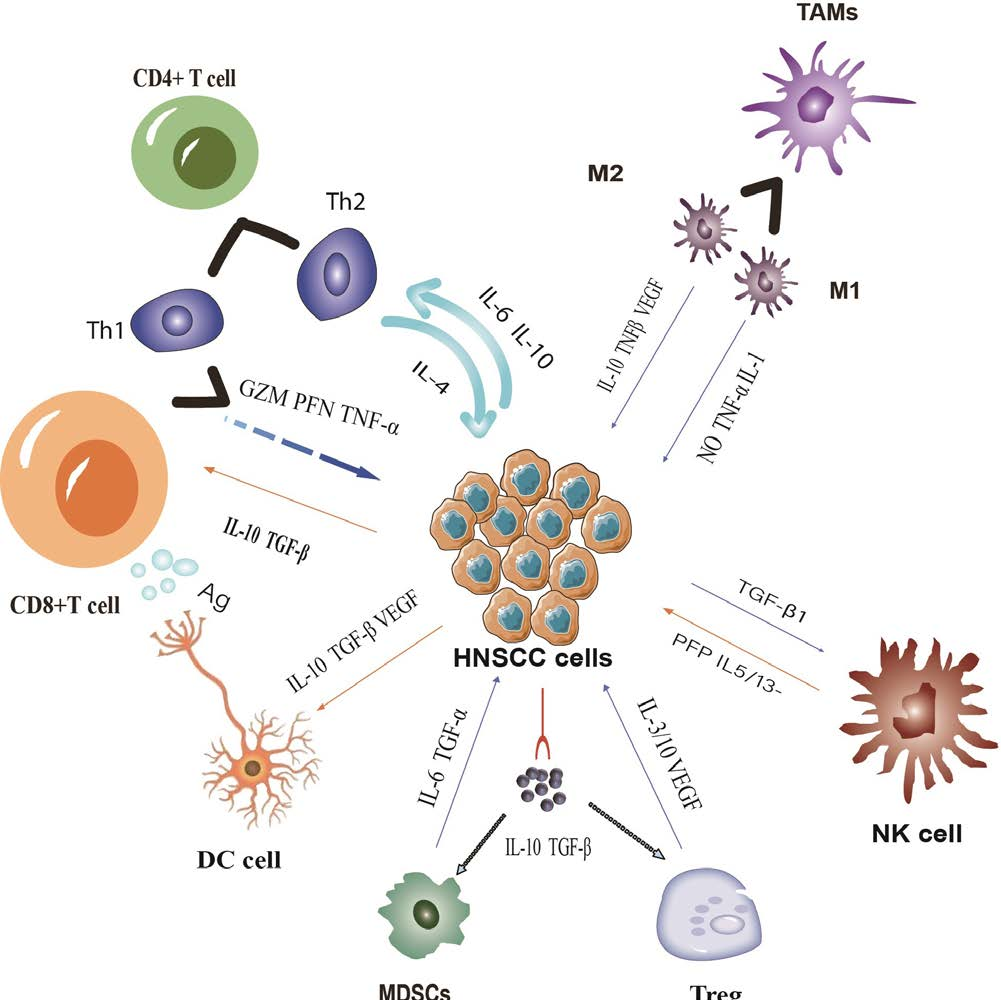
The mutual effect of a variety of different cell subsets, related factors and pathways can regulate the development of tumor cells in the tumor immune microenvironment of head and neck cancer.

Tumor microenvironment (TME) refers to the internal and external environment of tumor cells during the process of tumor development, which is composed of tumor cells and Extracellular matrix ECM. Tumor cells and their surrounding microenvironment constantly interact and evolve, and select traits that are conducive to tumor occurrence, invasion and metastasis in the course of tumor progression.^[6,7]^ In general, TME is an extremely complex environmental system, which can be roughly divided into immune microenvironments and non-immune microenvironments made up of stromal fibroblasts and tumor cells.^[8]^ The mechanism of immune microenvironment affecting tumor progression principally comprises promoting tumor angiogenesis and changing tumor biological characteristics. Not only can human immune system suppress or destroy cancer cells, but it is also able to screen tumor cells that are more suitable for the host microenvironment, and also create a suitable TME, with the aim of advancing tumor progression and regulating the activity of tumor stem cells.^[6,9,10]^ For the past few years, a great number of studies have manifested that in the TME of HNSCC, dysfunction of multiple immune killer cells and abnormal secretion of immune-related factors can induce immunosuppression and ultimately lead to the progression of HNSCC.^[11,12]^ The tumor immune microenvironment (TIME) is constituted by multiple distinct cell subpopulations including cancer-associated fibroblasts, macrophages, T cells, B cells, neutrophils, dendritic cells (DC), myeloid-derived suppressor cells (MDSC), natural killer cells (NK), mast cells, and so on. Tumor infiltrating lymphocyte (TIL) interacts with cancer cells and the extracellular matrix.^[13,14]^ Interactions among multiple cell subpopulations and different cytokines as well as pathways have a major role to play in mediating immune surveillance and controlling the occurrence and development of tumors (Fig. 1). Studies have revealed that T cell autoinhibitory receptors (also known as immune checkpoints) can act as momentous regulatory factors in the TME.^[15]^ In HNSCC patients, immune checkpoint is the basic mechanism leading to the escape of cancer cells. The high expression of immune checkpoint signals in the TME enables cancer cells to successfully get rid of the attack of immune cells.^[16,17]^ To date, it has been confirmed that the expression of immune checkpoint is significantly correlated with the stage and prognosis of some malignant tumors. This discovery makes immune checkpoint be used as an indicator for tumor intervention and detection, and interventions targeting immune checkpoints are expected to be new immunotherapeutic modalities.

This review mainly focuses on summarizing the classification and mechanism of action of TME cell subpopulations in HNSCC and discusses the opportunities and challenges faced by targeted immunotherapy. Aiming to grasp the important role of the TIME in HNSCC occurrence and progression, this study can offer a feasible direction for exploring multimodal therapeutic approaches combining immunotherapy, cytotoxic drugs, and molecular targeted drugs for HNSCC.

## 2. Immune cell populations in HNSCC

### 2.1. T cells

T-cell lymphocytes are considered as an important part of the immune system and consist primarily of CD3^+^T cells, CD4 ^+^T cells and CD8^+^T cells. CD3^+^T lymphocytes not only exist in resting lymphocytes, but also in activated lymphocytes. It was found that high density of CD3^+^TILs and CD8^+^TILs in the tumor center of HNSCC significantly improved the overall survival of patients. In addition, CD3^+^TILs in tumor invasion margins and CD8 + TILs in tumor centers are independent prognostic factors for oral squamous cell carcinoma (OSCC).^[18]^

Initial CD4^+^ T cells can differentiate into different subtypes of T cells (e.g., Th1, Th2, Th9, regulatory T cells [Treg] cells, etc) under varied conditions after receiving antigenic stimulation, and diverse CD4^+^ T lymphocyte subtypes have disparate or even opposite functions and mechanisms of action. Consequently, it is unclear that CD4 + T lymphocytes act on the TME.^[19]^ For instance, in patients with laryngeal cancer, disease-free survival and disease-specific survival are significantly prolonged in patients with high CD4 + and CD8 + TIL levels.^[20]^ And in a survival analysis of patients with oropharyngeal cancer, it was detected that the elevated proportion of CD4 + T/Treg cells was an independent factor for favorable prognosis of survival without distant metastasis.^[20]^ The above mentioned studies indicated that the immunomodulatory functions of different CD4 + T lymphocyte subpopulations are provided with evident differences and their role in HNSCC remains unknown, which are necessary to make a further research.

CD8^+^T cells are the dominant effector cells of anti-tumor immune response, which can bind to helper T1 cells and release perforin, granulozyme and tumor necrosis factor-α (TNF-α) to directly kill tumor cells and mediate anti-tumor immune response.^[21]^ However, the activity of CD8^+^T cells is suppressed to some extent in the TME. That is because there exist a myriad of different cytokines in tumor tissues, such as interleukin (IL-10), transforming growth factor-β (TGF-β) and so on that can inhibit the function of effector T cells. Among them, IL-10 can inhibit the conversion of T cells to cytotoxic effector T cells. TGF-β can inhibit the cytotoxic proliferation, differentiation or immune activity of T lymphocytes and NK cells, while TGF-β can also convert the cytotoxic effect of NK cells into a secretory effect, and secretory effector NK cells are able to produce a series of cytokines, such as vascular endothelial growth factor (VEGF) and IL-8 to promote tumor growth and angiogenesis.^[22]^

Treg have become a recently discovered subgroup of T lymphocytes whose dominant phenotypes includes CD4^+^, CD25^+^ and Foxp3^+^.^[23]^ Treg has active immunomodulatory function, which not only plays an important role in maintaining autoimmune tolerance, but also plays an indispensable part in infection, tumor, transplantation and autoimmune diseases.^[24]^ Treg can promote tumor proliferation by promoting tumor neovascularization and inhibiting anti-tumor immune activity. Studies have found that Treg is highly expressed in peripheral blood and tumor tissues of patients with head and neck cancer, lung cancer, breast cancer and other tumors, and is significantly correlated with poor prognosis of patients.^[25–27]^ Recent research indicated that Treg has been shown to cause immunosuppression by inhibiting the proliferation of effector T cells, regulating the production of related cytokines and suppressing the maturation of DC cells.^[28]^ Treg exerts immunosuppressive effects through multiple mechanisms of action in the development of tumor. As such, the study of multiple pathways, functions and regulatory mechanisms of Treg cells also provides new targets for immunotherapy of tumors.^[29,30]^

### 2.2. Tumor-associated macrophages (TAMs)

TAMs are one of the important immune cells for tumor infiltration and are differentiated from monocytes, which are classified into 2 subpopulations, that is, M1 and M2. Both M1 and M2 macrophages have high plasticity and they can be interconverted in response to changes in the TME or therapeutic intervention, with the proportion of each form determined by the type and concentration of different signals in the tumor environment.^[31–33]^ M1 macrophages, also known as classically activated type, exert anti-tumor effects mainly by secreting a great many factors such as carbon monoxide, TNF-α, and 1L-1. In contrast, M2 macrophages secrete a large number of cytokines such as IL-10, VEGF and TGF-β, which promote tumor cell proliferation, epithelial mesenchymal transition (EMT) (D) and angiogenesis. At the same time, it can inhibit anti-tumor immune response and also restrain the anti-tumor effect of M1.^[34,35]^

The research findings revealed that TAMs can promote EMT of HNSCC cells by secreting cytokines such as epidermal growth factor and TGF-β, and enhance the metastatic and invasive ability of HNSCC cells^[36]^; In addition, TAMs can induce the EMT process in HNSCC cells through the ERK1/2 signaling pathway, which may become a new therapeutic target for HNSCC. In oral cancer, TAMs expressing CD206 secrete epidermal growth factor, which in turn can promote invasion, metastasis and poor prognosis of OSCC.^[37]^ In nasopharyngeal carcinoma (NPC), NPC cell-derived basic fibroblast growth factor-2 causes vascular-associated pericytes to produce chemokine 14, which then induces the polarization of TAMs into M2 macrophages, thus promoting distant metastasis of NPC.^[38]^ It can be concluded that scores of studies have uncovered that increasing the density of TAMs can facilitate the progress of HNSCC, but it is urgent to further explore the specific mechanism and pathway of TAMs.

### 2.3. Myeloid-derived suppressor cells

MDSCs are viewed as immature MDSCs that are prevalent in cancer patients. Activated MDSCs can produce a large number of IL-6, TNF-α and other factors, which mediate inflammatory response and promote tumor progression.^[39]^ Studies have made it clear that peripheral MDSCs in lymphatic organs inhibit antigen-specific CD8 + T cells, while MDSCs in TME can control antigen-specific and antigen-nonspecific T cell function, leading to immunosuppression.^[40]^ In multiple tumor types, MDSCs are associated with tumor prognosis on account of their ability to inhibit cytotoxic CD8 cells by inducing production of IL-10, TGF-β, and Tregs^+^T cells, which in turn accelerates tumor growth and metastasis.^[41,42]^ In NPC cells, MDSCs enhance the expression of epoxy enzyme-2 (COX-2), which in turn activates the β-catenin/T cytokine 4 pathway, thus leading to EMT of NPC cells. And blocking COX-2 or MDSCs can effectively inhibit the invasion and metastasis of NPC.^[43]^ A studies was verified and demonstrated that lMP1-mediated glycolysis can regulate the production of IL-1β, IL-6 and GM-CSF in NPC through NOD-like receptor thermal protein domain associated protein 3 (NLRP3) inflammasome, COX-2 and phosphorylated *P*-65 protein (P-p65) signaling pathways in mice, thereby promoting MDSC amplification and further inducing tumor immunosuppression in NPC.^[44]^

### 2.4. NK and DC

DC are one of the major antigen-presenting cells (APC), whose role is to uptake, process and present antigens to CD4^+^T cells and CD8^+^T cells attempting to initiate immune responses.^[45,46]^ However, in tumor tissues, under the action of chemokine ligand 2 (CCL-2), DC will not only be affected by the interaction between tumor cells, but also become an immature DC phenotype with weak antigen-presenting ability under the inhibition of IL-10, TGF-β, VEGF and other factors, thus contributing to the immune escape of tumor.^[47]^ NK cells are derived from lymphoid stem cells in bone marrow and are the first line of defense against cancer cells. They can nonspecifically kill tumor cells and virus-infected lymphocytes without prior sensitization.^[48]^ NK cells have the ability to directly kill cancer cells, and the interaction between NK cells and other immune cells such as T cells in TME not only has a powerful anti-tumor effect, but also enhances the uptake of tumor antigen by DC, which can present antigen and activate T cell immunity.^[49]^ On the contrary, in HNSCC, NK cells and DC cells are in a suppressed state. Tregs in TME can suppress CD8 + T cells and NK cells directly or by secreting TGF-β and IL-10.^[50]^

However, in TME, NK cells are damaged by inhibitory cytokines along with an abnormal metabolic environment such as hypoxia, lack of application, and high lactate concentration, which can hinder CTL proliferation and cytokine production.^[51]^ Therefore, therapies that interfere with immune metabolism are important tools to improve the efficacy of NK cell dependent anti-tumor.

## 3. Immune escape and immune checkpoint

**Figure 2.**
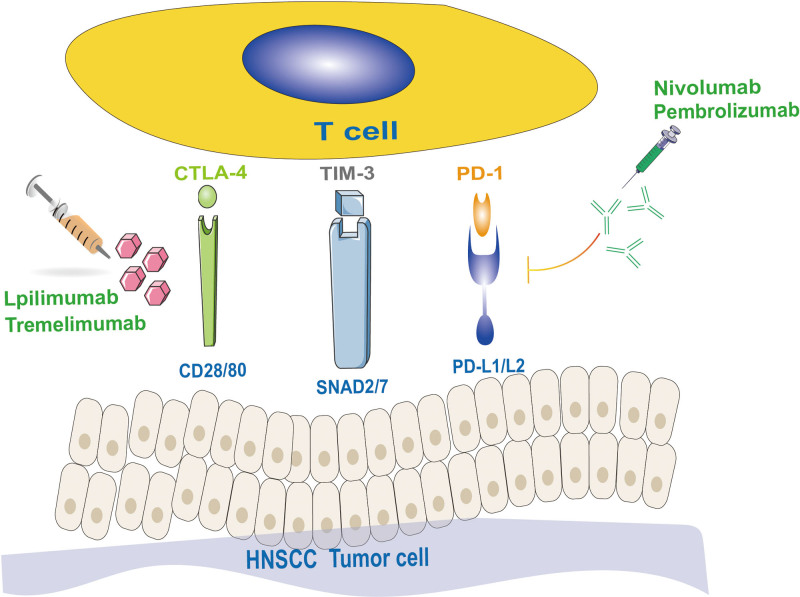
In HNSCC, it is proved that immune checkpoints such as TIM-3, PD-1 and CTLA-4 give rise to immune escape by affecting T cell function. In contrast, drugs targeting immune checkpoints (e.g., Nivolumab, Pembrolizumab, ipilimumab tremelimumab, etc) can tremendously be beneficial for effective therapeutic effects in tumors by blocking the interaction between the receptor and its ligand. HNSCC = head and neck squamous cell carcinoma, TIM-3 = T-cell immunoglobulin mucin-3.

Immune cells have a dual protective and tumor-promoting effect on the host, a role known as tumor immune editing, which can be simply classified as clearance, homeostasis and immune escape. Tumor immune escape refers to tumor cells modifying their own surface antigens and evading the body immune system to recognize and monitor and attack them through various ways such as suppressive immune cells, molecules and altering the microenvironment around tumor tissues, so as to continue to divide and proliferate.^[52]^ In HNSCC, immune checkpoint receptors are an important factor in controlling the immune system, limiting autoimmunity and regulating the inflammatory response, and are an essential mechanism, leading to the escape of cancer cells (Fig. 2). Studies found that various receptors such as lymphocyte activation gene 3, CTLA-4, T-cell immunoglobulin mucin-3 (TIM-3) and PD-1 have been detected to be expressed in T lymphocytes.^[53]^

### 3.1. Role of PD-1/programmed cell death ligand (PD-L1) in immune escape of HNSCC

Programmed death protein 1 (PD-1) served as one of the main immune checkpoints of T cells mediates immune escape by transmitting inhibitory signals to tumor-killer T lymphocytes through multiple pathways (see Table 1).^[64]^ PD-L1 is a transmembrane protein that is thought to be a co-suppressor of immune response. The immune checkpoint receptor PD1 expressed on T cells binds to PD-L1 in tumor cells, resulting in T cell dysfunction by reducing T cell receptor signaling pathways, cytokine production, cell migration, and promoting T cell differentiation into Tregs.^[65–67]^

**Table 1:** Immune escape mechanism of PD1 associated with HNSCC.

Tumor types	Mode of action	Quote
Esophageal cancer	miR-873 exerts a tumor suppressor effect by targeting DEC2 expressed in differentiated embryonic chondrocytes in esophageal cancer.	^[54]^
Nasopharyngeal cancer	AFAP1-AS1, which is positively correlated with PD-1 expression, promotes the invasion and metastasis of nasopharyngeal carcinoma by regulating actin cell keratin signaling pathway.	^[55,56]^
Esophageal cancer	High expression levels of PD-L1 and PD-L2 inhibit the immune effect of CD8 + T, thus inhibiting its tumor killing function.	^[57,58]^
Nasopharyngeal cancer	CircBART2.2 can promote immune escape from nasopharyngeal carcinoma by modulating PD-L1.	^[59]^
Nasopharyngeal cancer	EBV-miR-BART11 and EBV-miR-BART17-3p inhibit FOXP1 and PBRM1, respectively, and enhance the transcription of PD-L1, thus promoting tumor immune escape.	^[60]^
Nasopharyngeal cancer	Epstein-Barr virus-induced ectopic CD137 transfers CD137 from T cells to CD137L expressing antigen presenting cells, forming CD137-CD137L complexes for degradation and ultimately leading to immune escape.	^[61]^
Throat cancer	APE1 can regulate the expression of PD-L1 through the NF-κB signaling pathway, thereby modulating the immune response.	^[62]^
Throat cancer	In laryngeal cancer tissues, AEG 1 promotes PD-L1 activation at the transcriptional level, and miR-217 inhibits the metastasis and invasion of laryngeal cancer by blocking immunosuppressive signaling by inhibiting target gene AEG 1.	^[63]^

AFAP1-AS1 = LncRNA Actin filament associated protein 1 antisense RNA 1, APE = apyrimidinic endonuclease, CD137L = CD137 ligand, FOXP1 = forkhead box protein P1, HNSCC = head and neck squamous cell carcinoma, PBRM1 = polybrominated protein, PD-L = Programmed Cell Death ligand.

It has been shown that molecules including microRNAs (miRNAs) and long-stranded non-coding RNAs can regulate immune responses by interacting with PD-1/PD-L1. For example, miRNA-873 exerts tumor suppressive effects by targeting differentiated embryonic chondrocytes 2 expressed in esophageal cancer.^[54]^ Long-stranded non-coding RNA Actin filament associated protein 1 antisense RNA 1 is highly expressed in NPC and is positively correlated with PD-1 expression, which can promote cancer cell invasion and metastasis by regulating the expression of several small GTPase family members and molecules in the actin cell keratin signaling pathway.^[55,56]^ In esophageal cancer, PD-L1 and PD-L2 are highly expressed and suppress the immune effect of CD8^+^T. Conversely, blocking this pathway can restore the tumor-killing function of CD8 + T cells.^[57,58]^ The RNA CircBART2.2 encoded by Epstein-Barr virus is highly expressed in NPC cells, and has the function of regulating PD-L1 expression and suppressing T-cell immune function, so that CircBART2.2 can promote immune escape from NPC by regulating PD-L1.^[59]^ Some studies found that EBV-miR-BART11 and EBV-miR-BART17-3p, miRNAs encoded by EBV, can upregulate PD-L1 expression in EBV-associated NPC.^[60]^ Moreover, EBV-miR-BART11 and EBV-miR-BART17-3p can combine with the enhancer region of PD-L1 to promote tumor immune escape by targeting and inhibiting forkhead box protein P1 and polybrominated protein.

Based on such findings, it can thus be made a point that Epstein-Barr virus-induced ectopic CD137 expression is conducive to the evasion of immune surveillance in NPC. CD137 is a potent costimulatory receptor on activated T cells, which enhances anti-tumor immune response when CD137 levels are upregulated, while its negative feedback mechanism shifts CD137 from T cells to APC expressing CD137 ligand, forming CD137-CD137 ligand complex for degradation, accordingly, leading to immune escape.^[61]^ The results of a study further revealed that PD-L1 is overexpressed in laryngeal squamous cell carcinoma (LSCC) and hypopharyngeal squamous cell carcinoma tissues and is expressed at higher levels in vocal and subglottic compared to supraglottic LSCC tissues. Furthermore, the study suggested that upregulated co-expression of PD-L1 and human apurinic/apyrimidinic endonuclease 1 is a biomarker for LSCC and apyrimidinic endonuclease 1 can regulate PD-L1 expression through the NF-κB signaling pathway, therefore, which in turn regulates the immune response^[62]^; besides, in laryngeal cancer tissues, AEG 1 is equipped with the function that can promote the activation of PD-L1 at the transcriptional level, and thus miR-217 can inhibit the metastasis of laryngeal cancer by blocking immunosuppressive signal transduction and enabling patients to produce effective anti-cancer immune response aiming at suppressing target gene AEG 1.^[63]^

### 3.2. Expression of CTLA-4 in HNSCC

Cytotoxic T lymphocyte antigen 4 (CTLA-4), also referred to as CD152, is a transmembrane protein encoded by the C7X4-4 gene, which is mainly expressed in T cells. Under steady-state conditions, CTLA-4 is expressed in Tregs, and CTLA-4 is the target gene of Foxp3, the major regulator of Treg.^[68]^ It is concluded from mouse experiments that down-regulation of CTLA-4 expression levels can lead to Treg loss of function, and thus CTLA-4 can act as an immune checkpoint by regulating the function of Tregs.^[69]^ Studies have shown that CTLA-4 is highly expressed in CD8 + lymphocytes of laryngeal and nasopharyngeal cancers and is negatively correlated with patient survival.^[70–72]^ Both CTLA-4 and CD28 are transmembrane receptors that play opposite roles in mediating T cell activation. B7/BB1 is the cell surface protein of CD80. In HNSCC, CTLA-4 competes with CD28 to bind to the B7 family of molecules on APCs, inducing T cell dysfunction after binding to the B7 protein, thereby leading to immunosuppression.^[73,74]^ CTLA-4 can also prevent T cells from being further activated by binding to 2 ligands on the surface of APC, CD80 and CD86, and transmitting negative signals.^[75]^ Also, CTLA-4 combines with PI3K, resulting in phosphorylation of AKT, which is more likely to cause inactivation of pro-apoptotic factor BAD, and upregulation of anti-apoptotic factors Bcl-xL and Bcl-2, thus boosting tumor immune tolerance.^[76]^

### 3.3. Mechanism of action of TIM-3

TIM-3 is a member of TIM family, which is expressed in helper T1 cells, Th17, CD8 + T, DC, monocytes and other cells. TIM-3 usually mediates its inhibitory activity on immune cells through several ligands such as C-type galactose lectin-9 (Gal-9), phosphatidylserine (PtdSer), while when TIM-3 is expressed with PD-1 together, it will cause CD8^+^T lymphocyte depletion.^[77]^ Inhibition of TIM-3 in DC increases the accumulation of reactive oxygen species and activates NLRP3 inflammasome thereby lifting the potential for antitumor immunity.^[78]^ TIM-3 plays a dual role in tumor immunity and at the early stage of tumor formation and TIM-3 positive CD4 + T cells can secrete Interferon (IFN)-γ and promote anti-tumor effect, while TIM-3 + Tregs cells proliferate in the middle and late stage of tumor formation, so that it can block the function of effector T cells.^[79]^

A multivariate analysis of the findings in this study highlights that in HNSCC, TIM-3 expression levels are strongly associated with tumor size, lymph node metastasis and TNM stage, but they are not related with gender, age, tumor location, tumor differentiation, or history of smoking or alcohol consumption.^[80]^ Also, in patients with stage III and IV head and neck squamous cell carcinoma, overall survival (OS) and disease-free survival times are shorter in patients with high TIM-3^+^TIL infiltration than in those with low TIM-3^+^TIL infiltration. TIM-3 is expressed at significantly higher levels in NPC cells than in nasopharyngeal epithelial cells, and TIM-3 can promote tumor progression in NPC by mediating epithelial-mesenchymal transfer of human signal transduction molecule 7 (SMAD7), SMAD2 and zinc finger transcription factor 1 (SNAIL1).^[81]^

## 4. Advances in HNSCC immune microenvironment therapy

### 4.1. Immune checkpoint inhibitors

In recent years, immune checkpoint inhibitors have become a heated research issue in immunotherapy, which can play a wide and effective therapeutic role in a variety of tumors by blocking the interaction between inhibitory receptors and their ligands. In HNSCC, high expression levels of PD-1 and others can lead to shorter overall survival and recurrence-free survival, and are independent risk factors for death, treatment failure and local recurrence in NPC. Hence, anti-PD1/PDL1 monoclonal antibody is one of the crucial potential therapeutic targets.^[82]^ Nivolumab and pembrolizumab are IgG4 anti-PD-1 monoclonal antibodies that not only block co-inhibitory signaling pathways through the PD-1/PD-L1 axis, but also enhance K^+^ channel activity and CD8^+^T cell activity and response in HNSCC patients, ultimately improving overall patient survival.^[83–85]^ Studies found that in nasopharyngeal cancer, Pembrolizumab had an odds ratio of 22.2 percent for nasopharyngeal cancer and 77.8 percent disease control in patients.^[86]^ The anti-CTLA-4 ipilimumab and tremelimumab are also among the common immune checkpoint inhibitors. Blocking CTLA-4 leads to a decrease in Treg frequency and an increase in T cell function. In patients with HNSCC, ipilimumab enhances the activity of NK by decreasing the suppression of NK by Treg.^[87,88]^

### 4.2. Immunotherapy for TAM

TAMs are perceived as an important part of the TIME, and it is regarded as a hot research topic to use them as therapeutic targets. Immunotherapy strategies for TAMs are mainly to directly reduce the number of TAMs cells and change their biological function, namely, to induce the redifferentiation of Tams and weaken their function of promoting tumor progression.^[35,89,90]^ It was found that cancer cells further enhance the metastatic and invasive ability of cancer cells by secreting colony-stimulating factor-1 (CSF-1) to recruit and aggregate TAMs in the blood. Therefore, blocking the interaction of CSF-1 with its receptor results in a significant decrease in the number of TAMs and the proportion of M2 macrophages.^[91]^ CCL-2 interacts with chemokine receptor 2 (CCR2), a major pathway for replenishing TAMs from monocytes/macrophages, and disruption of the CCL-2-CCR2 pathway significantly reduce the number of TAMs in tumors, thereby curbing tumor growth and spread in some cancer types; CCL-2-neutralizing antibodies and small molecule inhibitors of CCR2 significantly inhibit tumor cell growth.^[92,93]^ Cancer-associated fibroblasts derived recombinant human chemokine 12 recruits M2 macrophages to infiltrate tumors, thus promoting the proliferation and migration of cancer cells in OSCC.^[94]^

### 4.3. Monoclonal antibody therapy

Monoclonal antibody is an essential targeted drug in tumor therapy. The combination of monoclonal antibody and the antigen on the surface of tumor cells can kill tumor cells, control the growth of tumor cells and delay the progression of the disease, so as to improve the prognosis of patients and prolong their survival time. Cetuximab is a representative drug of monoclonal antibodies against EGRF, which can be used in the treatment of recurrent/metastatic HNSCC. EGFR directly inhibits tumor cell proliferation, initiates complement-mediated tumor cell lysis, and directly kills tumor cells through antibody-dependent cell-mediated cytotoxicity.^[95]^ Cetuximab coated HNSCC cells have been shown to induce NK cells, promote DC cross-presentation effects and amplify EGRF-specific cytotoxic T cells.^[96]^ The monoclonal antibodies nivolumab and pembrolizumab, which target PD-1, and atezolizumab, which targets its ligand PD-L1, have shown good results in metastatic HNSCC.^[97]^ According to a phase 2 clinical trial, the study results showed that a new adjuvant regimen of Nivolumab alone or Nivolumab plus ipilimumab is safe and effective in OSCC.^[98]^

### 4.4. Combination immunotherapy

There are specific situations in which combination immunotherapy can be more beneficial to patients than monotherapy in that the TME of HNSCC consists of multiple cells, receptors, and signaling pathways. The present studies have proved that the anti-tumor effect of immune checkpoint inhibitors in patients with HNSCC is enhanced after radiotherapy/chemotherapy treatment, as evidenced not only by increased infiltrative activity of peripheral blood CD8^+^T cells, but also by increased numbers of suppressor Treg cells and PD1-positive T cells.^[99,100]^ The anti-EGFR monoclonal antibody Cetuximab in HNSCC, when used in combination with immune checkpoint inhibitors, not only induces a specific immune response, but also alters the expression of immune checkpoint receptors on TILs, so that it can further enhance the immune response to tumors.^[101]^ The study showed that Cetuximab combined with chemoradiotherapy results in better outcomes, longer survival and lower mortality than chemoradiotherapy alone, in patients with advanced HNSCC.^[102]^ Anti-PDL1 monoclonal antibody Durvalumab is more effective than a combination regimen with tremelimumab alone in relapsed/metastatic HNSCC.^[103]^

As an important means of combined immunotherapy for HNSCC, drug-related adverse events are not uncommon. However, these serious adverse events can be controlled by suppressing the immune system. Therefore, more studies and data are needed to find the optimal regimen and dosage for combined immunotherapy in order to avoid over-stimulating or suppressing the immune system as much as possible, so that combined immunotherapy can be better applied in the clinic.^[104]^

## 5. Conclusion

As is discussed above, in short, the composition of each immune cell subpopulation, cytokine interactions and immune checkpoint-related pathways in HNSCC have vitally important effect on the occurrence, development and immune escape of HNSCC. Tumor cells tends to evade immune surveillance through a variety of mechanisms to promote the proliferation and metastasis of tumor cells.

As the mechanism of the immune microenvironment of HNSCC has been constantly studied, systematic therapeutic strategies have been continuously improved. However, it is noteworthy that the ensuing lack of research on drug resistance, predictive biomarkers, and the associated combination immunotherapy will cause relevant adverse effects. Therefore, more basic and clinical studies are needed to explore the optimal treatment modalities and seek a reasonable and appropriate therapeutic doses in order to effectively improve the prognosis of HNSCC patients and enhance their quality of life.

## Author contributions

**Conceptualization:** Qichao Hong, Zhonglin Mu.

**Investigation:** Qichao Hong, Shun Ding.

**Writing – original draft:** Qichao Hong.

**Writing – review & editing:** Shun Ding, Chengliang Xing.
